# Evidence for Molecular Mimicry between SARS-CoV-2 and Human Antigens: Implications for Autoimmunity in COVID-19

**DOI:** 10.1155/2024/8359683

**Published:** 2024-08-31

**Authors:** Andrea Arévalo-Cortés, Daniel Rodriguez-Pinto, Leonardo Aguilar-Ayala

**Affiliations:** ^1^ Faculty of Human Health Universidad Nacional de Loja, Loja 110103, Ecuador; ^2^ Department of Health Sciences Faculty of Health Sciences Universidad Técnica Particular de Loja, Loja 110108, Ecuador

## Abstract

As for other viral diseases, the mechanisms behind the apparent relationship between COVID-19 and autoimmunity are yet to be clearly defined. Molecular mimicry, the existence of sequence and/or conformational homology between viral and human antigens, could be an important contributing factor. Here, we review the accumulated evidence supporting the occurrence of mimicry between SARS-CoV-2 and human proteins. Both bioinformatic approaches and antibody cross-reactions have yielded a significant magnitude of mimicry events, far more common than expected to happen by chance. The clinical implication of this phenomenon is ample since many of the identified antigens may participate in COVID-19 pathophysiology or are targets of autoimmune diseases. Thus, autoimmunity related to COVID-19 may be partially explained by molecular mimicry and further research designed specifically to address this possibility is needed.

## 1. Introduction

The coronavirus disease 2019 (COVID-19) pandemic has caused more than 7 million deaths globally [1]. This disease is due to infection by severe acute respiratory syndrome coronavirus 2 (SARS-CoV-2), and the global response included the rapid development of vaccines that were massively administered to the human population. In spite of all the basic science knowledge and medical advances of our era, COVID-19 pathophysiology and its complications are still poorly understood, including the immune response generated by vaccination [2, 3]. In this context, autoimmune phenomena are of great interest because many of the alterations observed in COVID-19 may be derived from immune system dysregulation that leads to autoimmunity, and autoimmune pathology has been observed after the disease and vaccination [4, 5]. For this reason, here we review studies that address one potential mechanism that explains the relationship between COVID-19 and autoimmunity: molecular mimicry. This term describes the existence of homologous sequences and/or structures in human and SARS-CoV-2 antigens and its study may shed light on the appearance of autoimmune pathology after COVID-19 or vaccination [6].

The aim of this review is to summarize the current knowledge on the impact of molecular mimicry on the pathophysiology of COVID-19 and autoimmune diseases that appear after the disease or vaccination, establishing the magnitude of this phenomenon and highlighting the type of evidence that exists so far to support it. First, we will start with a brief synopsis of the role of molecular mimicry as an autoimmune trigger. Then, we will summarize evidence of molecular mimicry between SARS-CoV-2 and human antigens, and finally, the implications of these findings in relation to COVID-19 disease will be discussed.

## 2. Molecular Mimicry in the Genesis of Autoimmunity

A healthy immune system must recognize pathogenic microorganisms to generate an immune response that halts serious disease and generates memory able to eliminate the microorganism in a subsequent infection. However, it has been noted that during this normal process, events that contribute to the breakdown of tolerance to self-antigens may occur [7]. Molecular mimicry refers to the presence of homology between human antigens and antigens from microorganisms, which results in the attack of human tissues by the effector mechanisms correctly generated to combat the pathogen [8]. Together with other mechanisms, such as the activation of bystander lymphocytes and secretion of superantigens (bacterial proteins that can activate a large number of T-cell clones by binding the TCR irrespective of its specificity [9]), mimicry is considered an important trigger of autoimmunity associated with infections [10–12].

This phenomenon is due to the existence of sequences with a high degree of identity within epitopes from humans and microorganisms. An epitope is defined as the portion of the antigen that binds lymphocyte antigen receptors, namely, the T-cell receptor (TCR) in T cells and the B-cell receptor (BCR) in B cells. Mimicry can result from the presence of identical amino acid sequences in human and pathogen proteins that form linear epitopes that can activate both T cells and B cells. Nonlinear or conformational epitopes that arise when an amino acid chain acquires its secondary and tertiary structures can also determine mimicry for any type of antigen (carbohydrate, lipid, protein, or nucleic acid) and may lead to autoreactive B cell activation [8, 10]. Finally, posttranslational modifications that further alter antigen structure have also been shown to influence the occurrence of molecular mimicry [13].

Four criteria must be met to establish if an antigen from a microorganism is a contributing factor for a particular autoimmune pathology due to molecular mimicry: (1) determine there is homology between antigen sequences and/or structures, (2) show cross-reaction between human antibodies or TCRs and the antigen, (3) establish an epidemiological relationship between the autoimmune pathology and the infection, and (4) reproduce the autoimmune pathology in an animal model after administration of the antigen [11, 14]. Even though a high frequency of homology between human and pathogen antigens exists, it has been very difficult to meet all these criteria in most cases of mimicry. This is due to several factors, among them that the methods to study the TCR repertoire in humans are very complex, that the latency period for the appearance of an autoimmune disease after an infection is often very long, and that there is no adequate animal model for most autoimmune diseases [8, 11].

Despite these difficulties, clear examples of molecular mimicry that contribute to autoimmunity have been described. The first one was reported more than 70 years ago between streptococcal protein M and antigens of human cardiac tissue, a cross-reaction that causes rheumatic fever. It is also known that Guillain–Barré syndrome can be ignited by diverse infections, including many viruses and the bacteria *Campylobacter jejuni*. It has been demonstrated that a superficial lipooligosaccharide from this bacterium has structural homology to GM1 ganglioside from human myelin. Antibodies directed to this antigen are able to induce the disease in an animal model [15, 16]. Another example is the Epstein–Barr virus antigen nuclear antigen-1 (AN-1) that has identical sequences to several human antigens that are related to autoimmunity, such as myelin basic protein in multiple sclerosis, and antigens Sm and Ro in systemic lupus erythematosus and Sjögren's syndrome. These autoimmune diseases have been epidemiologically associated with infection by the virus and in multiple sclerosis to the MHC-II allele HLA-DRB1 ∗ 15 : 01, which is able to present peptides from AN-1. Molecular mimicry has been shown for several other viruses, including enteroviruses, herpes simplex, hepatitis B, and influenza viruses [11].

### 2.1. Bioinformatic Evidence of Molecular Mimicry between Linear Epitopes of SARS-CoV-2 and Human Antigens

Most studies that have provided evidence of molecular mimicry between SARS-CoV-2 and human antigens use bioinformatic methods to find homologous sequences in proteins from both organisms. The most frequent procedure consists of using viral protein sequences that have been deposited in public databases and dividing them into short peptides ranging from 5 to 9 amino acids, because these sizes are the shortest ones considered to be immunogenic. To generate all possible peptides from the viral protein sequence, peptides that overlap by one amino acid are generated. Then, the presence of the exact sequence from these peptides in human proteins is determined using bioinformatic tools. Finally, the identical sequences found are analyzed in the immune epitope database (IEDB, https://iedb.org), which contains epitopes validated as immunogenic by experimental methods, as they can activate T cells, B cells, or both. This step is vital to demonstrating the potential of molecular mimicry resulting in a pathologic immune response [17–36].

Twenty studies have found evidence of molecular mimicry using this strategy, with a very wide range of proteins analyzed. On the virus side, eight studies [15, 18, 21–24, 28, 29] analyzed sequences from the whole SARS-CoV-2 proteome; one analyzed eight proteins [35], while seven [18, 21, 22, 27, 29, 32, 34] only searched for sequences from the spike protein. An additional four studies considered all epitopes from SARS-CoV-2 that are validated in IEDB as immunogenic [19, 28, 33, 36] (Tables 1 and 2).

Regarding human proteins, 14 studies are focused on a specific organ system, molecule, or pathology that is of the authors' interest (Table 1). In six of them [17, 22–24, 29, 33], the number of human proteins analyzed is very small (between one and five), but nonetheless, they all find occurrences of shared sequences between SARS-CoV-2 and the human antigens. The proteins considered in the other eight studies [18, 20, 21, 30, 31, 34–36] range from 10 to 92. While all of them found instances of molecular mimicry (between two and 169), the highest amount was found in a study that compared the sequences of the whole viral proteome with several human plasma proteins [31].

When the strategy included the complete human genome (five studies [19, 25–28]) or all of the human epitopes found in IEDB and validated as immunogenic (one study [32]), a higher magnitude of occurrences was found, with the study by Marino Gammazza et al. [25] reporting 3781 shared peptides between SARS-CoV-2 and human proteomes. The only other study comparing the whole proteome of both organisms [26] only reported 33 shared peptides. This large disparity is probably due to the use of peptides of eight and nine amino acids in the latter study, which results in a stricter search in comparison to the use of contiguous segments with a length of six amino acids or more in the former. Taken together, the results from these 20 reports indicate that a significant amount of molecular mimicry between SARS-CoV-2 and humans occurs at the protein sequence level.

It is important to highlight that several studies provide detailed calculations of the probability of viral sequences occurring in the human proteome by chance, which is extremely low, in the order of one in 64 million. The magnitude of occurrences of mimicry is much higher than those expected by chance, and it is proposed that it is due to the evolutionary relationship of both organisms [19, 27].

In addition, several studies use proteomes of other viruses or organisms as controls to validate the specificity of the findings to SARS-CoV-2. For example, Kanduc and Shoenfeld proved that mimicry between SARS-CoV-2 proteins and human surfactant was not the same as that found for HCoV-229E, a coronavirus that infects humans but causes a common cold. In that same study, proteins from viruses that do not cause respiratory disease, such as rabies, rubella, hepatitis C, and parvovirus B19 were also included; no instances of mimicry were found for those viruses in the human proteome. The proteome of the fungi *Pneumocystis jirovecii*, an important cause of pneumonia, was also included, and within it, they found 13 mimicry occurrences identical to those of SARS-CoV-2 proteins, out of a total of 24 [21]. In another study, Root-Berstein showed a significantly higher degree of mimicry between SARS-CoV-2 proteins and human proteins related to the coagulation and complement systems (169 instances) in comparison to other six viruses that presented only 36.2 instances on average. This author also analyzed the proteomes of several bacteria related to coagulopathies in which he found a pattern of mimicry similar to the one observed for SARS-CoV-2 [31]. Finally, Kanduc and Shoenfeld analyzed the SARS-CoV-2 spike sequence not only against the human proteome but also those of eight other mammals: mouse, rat, cat, dog, rabbit, chimpanzee, gorilla, and macaque. They found a high magnitude of mimicry only for mice and humans, a lower degree of mimicry (approximately half) for rats, and almost no mimicry for the other six species [27]. The inclusion of all these controls supports the notion that the molecular mimicry described is specific to SARS-CoV-2 and may be related to pathological conditions.

Two of the studies focus on special sequences from the spike protein. Anand et al. only searched for the occurrence of mimicry for a sequence of eight amino acids that is recognized specifically by a protease to allow the cleavage of the protein, a required step for SARS-CoV-2 entry into host cells. The sequence was found in a sodium channel called ENaC that also needs to be cleaved by proteases to function correctly. Thus, mimicry was proposed as a mechanism for altered ENaC function during SARS-CoV-2 infection. [29]. In another study, Talotta focused on the sequence of the SARS-CoV-2 spike protein that is codified by the Pfizer BNT162b2 vaccine and analyzed the epitopes found to have mimicry to determine if they could bind human MHC molecules. He found mimicry with five human proteins in epitopes that bind to MHC alleles associated with autoimmune diseases. Although experimental evidence linking the administration of the vaccine to immune responses to the implicated antigens is needed, these results raise a possible mechanism for this vaccine to contribute to the appearance of autoimmunity [32].

One study compared DNA sequences between SARS-CoV-2 and human genomes instead of protein sequences. It focused on retroelements and found identical sequences up to 35 bases long which coded for immunogenic epitopes found in IEDB. This finding is relevant because retroelements code for enzymes that can modify DNA and their regulation has been found to be altered in COVID-19, as well as in systemic autoimmune diseases [37].

### 2.2. Bioinformatic Evidence of Molecular Mimicry between Nonlinear Epitopes of SARS-CoV-2 and Human Antigens

It is well known that autoreactive B cell clones as well as the autoantibodies they produce recognize nonlinear or conformational epitopes that arise after protein folding and are constituted by noncontinuous regions of the amino acid chain that fold into secondary and tertiary structures [38]. Therefore, the hypothesis that molecular mimicry can trigger autoimmunity after SARS-CoV-2 infection requires the evaluation of nonlinear epitope similarity in addition to linear epitopes. Although bioinformatic methods to fulfil this need are more sophisticated and less common, three studies approached the search for molecular mimicry by generating models of the three-dimensional structure of SARS-CoV-2 proteins and comparing them to their human counterparts. Kakoulidis et al. used a novel technology called Machaon to compare the structure of SARS-CoV-2 spike protein to human proteins and established molecular mimicry in several proteins related to the immune response and some antigens relevant to autoimmunity [39]. Likewise, O'Donaghue et al. generated three-dimensional models of viral proteins and found that approximately 6% of the SARS-CoV-2 proteome has molecular mimicry with human antigens. These authors propose that viral proteins that mimic host proteins may sequester molecules that are needed for fundamental processes such as protein synthesis and the immune response, contributing to pathology [40]. Finally, Gutman et al. created three-dimensional models of shared peptides between SARS-CoV-2 and human antigens related to demyelinating diseases, finding significant overlap among them [35]. These three studies represent an additional level to establish mimicry and show that the application of such methods may unravel important relations between nonlinear epitopes from viral and human proteins.

### 2.3. Evidence of Molecular Mimicry between SARS-CoV-2 and Human Antigens by Antibody Cross-Reaction

The determination of cross-reaction between antibodies specific for SARS-CoV-2 antigens with human antigens is another method used to establish molecular mimicry that also reveals the existence of shared conformational epitopes. In addition, it provides evidence based on laboratory assays that complement the data acquired through bioinformatics. Importantly, in this interaction, proteins are used after posttranslational modifications, providing an additional structural element that may manifest as mimicry between human and viral antigens. Two studies used commercially available monoclonal antibodies against SARS-CoV-2 spike and nucleoprotein, while a third study used an anti-spike antibody obtained by the authors using hybridoma technology. The three studies evaluated cross-reaction with human antigens using enzyme-linked immunosorbent assays (ELISAs), testing multiple human proteins that are recognized as important targets in autoimmune responses. Results from two of the studies, which used a large antigen panel (more than 100 total antigens) revealed a significant number of cross-reactions: the antispike antibody reacted with 36 human antigens, while the antinucleoprotein antibody reacted with 32. After comparing the signal generated by the cross-reaction with that of the viral antigens, it was determined that 10 human antigens had high-affinity interactions with the antibodies, while the rest were weaker interactions, but nonetheless significant [41, 42]. The third study focused on antigens that play a role in iron metabolism and found cross-reaction with lactoferrin and transferrin [43]. These studies with antibodies show that the mimicry found between SARS-CoV-2 and human antigens with bioinformatic methods can be translated into interactions with the immune system that can result in pathology.

### 2.4. Possible Role of Molecular Mimicry in Cytotoxic T-Cell Autoimmune Responses

The activation of autoreactive CD8 T cells due to molecular mimicry has also been documented, although in less magnitude than for antibody responses. Reports of cytotoxic T cells in type 1 diabetes, psoriasis, aplastic anemia, and autoimmune hepatitis activated by epitopes shared between recognized autoantigens and proteins from Coxsackievirus, Streptococcus, Epstein–Barr virus, and hepatitis C virus, respectively, illustrate that the possibility of CD8 T cell activation due to mimicry is an important event in several autoimmune diseases [44–47]. Therefore, it is an intriguing possibility in SARS-CoV-2 infection, since the activation of this arm of the immune response is particularly important for defense against viruses. Accordingly, studies using immunoinformatic methods have reported viral epitopes that can bind to MHC-I molecules and are homologous to human autoantigens. For example, Karagöz et al. found several epitopes from the SARS-CoV-2 E protein that mimic retinal human proteins that bind MHC-I [48], while An et al. found the same in viral proteins that mimic human poly (ADP-ribose) polymerases [49]. It is therefore necessary to consider cytotoxic T cells as an additional potential effector mechanism for autoimmunity triggered by molecular mimicry.

### 2.5. Impact of Molecular Mimicry in COVID-19 Pathophysiology

SARS-CoV-2 is a single-chain RNA-enveloped virus that expresses the spike glycoprotein on its surface. This protein has a receptor-binding domain (RBD) that binds angiotensin-converting enzyme 2 (ACE2) expressed in several cell types of the human host, most notably the respiratory epithelium. This allows the virus to enter the cells and start its replication, which may lead to pathology. In most cases, the disease is mild and resembles other viral infections with fever, malaise, and dry cough. More severe cases present with shortness of breath and hypoxemia and some patients with the most severe presentations develop acute respiratory distress and multiorgan failure, with involvement of multiple systems including respiratory, cardiovascular, renal, digestive, neurologic, and hematologic [2, 3].

The pathophysiological mechanisms behind these clinical manifestations are complex and still not entirely understood. Since immune system dysregulation has been shown to be a fundamental component in the generation of severe pathology, it has been proposed that autoimmune phenomena may contribute to excessive inflammation and other disease manifestations [50–52]. Accordingly, several similarities have been described between immune phenomena that occur in autoimmune pathology and SARS-CoV-2 infection. First, there is excessive secretion of proinflammatory cytokines and chemokines such as interleukin-1 (IL-1), IL-6, IL-8, IL-18, CXCL10, and CCL2, many of which have been associated with disease severity, both in COVID-19 and autoimmunity. In addition to this cytokine storm, the activation and infiltration of immune cells follow a similar pattern in both pathologies, with excess of activated macrophages with hemophagocytosis, and neutrophilia with increased presence of neutrophilic extracellular traps. These shared characteristics may reflect common pathophysiological mechanisms in both diseases [4, 53, 54].

The hyperinflammatory state that characterizes COVID-19 has devastating systemic consequences and can lead to death [50]. In relation to this, three studies propose that molecular mimicry may lead to the autoimmune attack and ensuing malfunction of systems that normally keep the inflammatory response in check. The studies by Churilov et al. focus on the adrenal system, as they find mimicry between SARS-CoV-2 antigens and 21-hydroxylase, adrenocorticotropic hormone (ACTH) receptor, angiotensin II receptor, and proopiomelanocortin, proteins that play critical roles in the functioning of the hypothalamic-pituitary-adrenal axis. An autoimmune attack against them would diminish their role in the regulation of the inflammatory response contributing to the development of hyperinflammation [18, 33]. Likewise, Gamazza et al. found mimicry with several proteins that belong to the parasympathetic nervous system, which is known to have an important modulatory function in the inflammatory response. Therefore, an autoimmune response against these antigens may also favor hyperinflammation [20]. In conclusion, the mimicry between viral antigens and human proteins that inhibit inflammation may have the potential to contribute to a hyperinflammatory state in COVID-19.

Other studies found mimicry with antigens related to the respiratory system, one of the most affected in severe COVID-19. Kanduc and Shoenfeld found 24 viral protein sequences in proteins from human surfactants [21]. Lucchese and Flöel also found mimicry in the respiratory system, in their case with the proteins disabled homolog 1 (DAB1), apoptosis-inducing factor 1 (AIFM1), and Surfeit locus protein 1 (SURF1), which participate in the functioning of the respiratory pacemaker in the central nervous system. Changes in the function of these proteins have been previously described in respiratory diseases [23]. Finally, Anand et al. found mimicry with a sequence that guides proteolytic cleavage of the human epithelial sodium channel (ENaC). This protein has been associated with respiratory pathology because it is critical for the homeostasis of the liquid surface of the airways [29]. These studies propose several different mechanisms by which an autoimmune response due to molecular mimicry can generate respiratory alterations in COVID-19.

In a similar fashion, coagulopathies are a component of COVID-19 and mimicry could have an impact. Root-Berstein showed molecular mimicry between SARS-CoV-2 antigens and proteins from the coagulation system, such as platelet phosphodiesterase, factor IX, factor X, Von Willebrand factor, and disintegrin-like metalloproteinase with thrombospondin motif type 1 (ADAMTS)-13 protease. Based on these findings, the author proposes that mimicry may contribute to the hypercoagulability state. Interestingly, the author also found mimicry with antigens from bacteria that commonly coinfect patients with COVID-19 and are known to alter coagulation, suggesting there could be synergy in the effect of mimicry from various microorganisms [31].

Anosmia is a common manifestation of COVID-19 which may also be related to molecular mimicry. Three different studies found identical sequences between SARS-CoV-2 proteins and eight human olfactory receptors [19, 24, 55]. This implicates mimicry as a potential mechanism for olfactory alterations in COVID-19. Finally, retinal involvement in COVID-19 has been reported [56, 57], and the presence of the ACE2 receptor in the retina [58] makes the contribution of autoimmunity through mimicry a possibility. Thus, the finding by Karagöz et al. of mimicry between retinal proteins and the viral E antigen is important and warrants further exploration [48].

### 2.6. Impact of Molecular Mimicry in Post-COVID-19 Autoimmune Disease

Like other viruses, SARS-CoV-2 has been implicated as a trigger of autoimmunity, since there have been many reports of autoimmune diseases that present after the infection. One of these pathologies is Guillain–Barré syndrome, which has been described post-COVID-19 in several countries from Europe and Asia, as well as the United States. This disease is associated with autoantibodies against myelin that appear after many infections but are yet to be found after COVID-19. In the nervous system, multiple sclerosis and neuromyelitis optica have also been reported [59–61]. Other autoimmune diseases that have been observed after disease caused by SARS-CoV-2 include autoimmune thrombocytopenic purpura, autoimmune anemia, systemic lupus erythematosus, and a pathology similar to the systemic vasculitis Kawasaki disease. In addition, thrombotic phenomena have been observed in patients with antiphospholipid antibodies, such as deep vein thrombosis and pulmonary embolism [53, 62, 63].

Furthermore, the presence of autoantibodies in COVID-19 patients has been amply reported, and its occurrence has been found to be significantly higher than in healthy controls. They include antibodies found in systemic autoimmune diseases such as antinuclear antibodies (ANAs), antineutrophilic cytoplasm antibodies (ANCAs), antiphospholipid, and anti-Ro. Importantly, antiphospholipid antibodies have been associated with an antithrombotic state. Furthermore, type-I interferon-neutralizing autoantibodies have been detected in COVID-19; it was shown that these antibodies are associated with severe pathology in an animal model [62, 64–67].

Although these observations provide a temporal connection between immune responses to SARS-CoV-2 antigens and autoimmunity, possible mechanisms behind the cause, including molecular mimicry, have not been defined. However, in the studies reviewed here, multiple instances of molecular mimicry between the SARS-CoV-2 proteome and human antigens that are recognized markers of autoimmune disease have been established. Important examples include thyroglobulin, thyroid peroxidase, thyroid-stimulating hormone (TSH) receptor, glutamic acid decarboxylase, myelin basic protein, myelin oligodendrocyte glycoprotein, mitochondrial antigen, nuclear antigen, S100B, kisspeptin receptor (KISSR), cytochrome P450 2D6, histone H1.0, myosin, and heat shock proteins. These antigens have been associated with a wide range of systemic and local autoimmune pathologies, for example Hashimoto's thyroiditis, Graves' disease, type 1 diabetes, systemic lupus erythematosus, rheumatoid arthritis, Sjögren's syndrome, hypogonadotropic hypogonadism, autoimmune hepatitis, celiac disease, primary biliary cholangitis, ulcerative colitis and Guillain–Barré syndrome [17–19, 25, 26, 30, 35, 36, 39–42, 55]. This finding indicates that, as for other viruses, molecular mimicry is a potential trigger for autoimmunity after SARS-CoV-2 infection.

Autoimmune diseases have also been reported after vaccination for SARS-CoV-2 [5, 68]. For example, a series that reported information from four sources determined an incidence of approximately one case per million doses for thrombocytopenia secondary to mRNA vaccines. It is presumed that many of these cases may be autoimmune thrombocytopenic purpura triggered by vaccination, while other cases have been classified as a new entity: vaccine-induced immune thrombotic thrombocytopenia (VITT). However, the number of cases of thrombocytopenia is not higher than expected to appear in the general population. Cases of autoimmune hemolytic anemia, aplastic anemia, and antiphospholipid syndrome have also been documented but at extremely low frequencies. Nine cases of autoimmune hepatitis secondary to mRNA and DNA vaccines have been reported, with autoantibodies and typical biopsies that were treated successfully with steroids. In addition, a few cases of Guillain–Barré syndrome were presented one to three weeks after vaccination with a good prognosis and a favorable response to intravenous immunoglobulin [5].

Even though these examples of postvaccination autoimmunity have been reported with low incidence, they raise the possibility that viral antigens administered in the vaccine can trigger the loss of tolerance, with molecular mimicry playing an important role. It is important to highlight that Talotta's study detected sequence identity between antigens that can initiate autoimmunity in humans and the protein codified by the Pfizer-BioNTech mRNA vaccine [32]. Furthermore, instances of sequence identity were also found between the viral vector of the AstraZeneca COVID-19 vaccine, adenovirus 5, and several human plasma proteins [31]. These observations allow us to widen the potential mechanism to postvaccination autoimmune phenomena, which have also been reported. Vaccines are administered massively to healthy individuals and thus must be completely free of serious side effects; due to this, the continued study of the potential role of SARS-CoV-2 vaccines in molecular mimicry is warranted, especially in light of similar issues with autoimmunity that have arisen with other viral vaccines such as influenza, hepatitis B, and papillomavirus [69].

## 3. Conclusions

The studies reviewed here show that molecular mimicry between SARS-CoV-2 and human antigens occurs in a significant quantity. Its presence has been revealed by several methods, in most cases bioinformatic: protein or DNA sequence alignment and comparison of three-dimensional structure models. These methods have been complemented by studies of antibody cross-reactions, which constitute a validation of mimicry in the laboratory. Together, these results prove the existence of mimicry between the causative agent of COVID-19 and human antigens.

Given this phenomenon, it is important to establish the impact it may have on the disease, its complications, and vaccination. Mimicry has been identified for molecules that participate in COVID-19 pathophysiology, with possible mechanisms affecting several aspects of the disease, including hyperinflammation, respiratory function, hypercoagulability, and anosmia. Mimicry was also identified with antigens that are relevant in human autoimmunity, which can present both after COVID-19 and vaccination. Thus, the potential for mimicry to contribute to the disease and associated autoimmune pathology is evident, as well as its possible role in postvaccination autoimmune manifestations.

The evidence related to molecular mimicry between SARS-CoV-2 and human antigens generates challenges for COVID-19 research. Studies designed specifically to assess the role and possible alterations by autoimmune attack of the implicated human antigens in the disease are needed. Likewise, it is vital to develop protocols to determine if immune responses against these antigens have started in individuals with a high risk of developing autoimmunity who have suffered from SARS-CoV-2 infection. Finally, molecular mimicry should be considered when designing new vaccines or improving existing ones, ideally eliminating all viral sequences that are found in human antigens from the vaccine antigens. This would improve vaccine safety by eliminating a factor that may lead to autoimmunity.

It is worth noting that bioinformatic analysis of protein homology as well as *in silico* simulation of immune responses (termed immunoinformatics) have notable limitations related to the predicting accuracy of the methods. As a result, the translation of the information obtained by these methods to both *in vitro* a *in vivo* experiments does not always result in verification of the working hypothesis. However, the abundant information produced by bioinformatics presented in this review will provide a starting point to guide immunologists and clinicians who study autoimmune phenomena in the context of SARS-CoV-2 infection in their search for evidence of mimicry as a contributing mechanism. In regards to vaccine design, immunoinformatics has not been efficient at providing candidate antigens [70] but should be more valuable in providing information that leads to removing sequences from vaccines for safety reasons.

## Figures and Tables

**Table 1:** Bioinformatic studies addressing mimicry between SARS-CoV-2 and human proteins focused on a limited number of human proteins.

Focus	Human proteins	SARS-CoV-2 proteins	Shared peptides	Reference
Autoimmune hemolytic anemia	1	Spike	1	[22]
ENaC sodium channel	1	Spike	1	[29]
Hypothalamic system	2	Whole proteome	8	[17]
Respiratory pacemaker	3	Whole proteome	3	[23]
Anosmia and leukopenia	3	Whole proteome	3	[24]
Adrenal glands	5	Immunoepitopes^∗^	9	[33]
Demyelinating diseases	10	8 viral proteins	14	[35]
Endocrine glands	16	Spike	14	[18]
Parasympathetic nervous system	22	Whole proteome	21	[20]
Coagulopathies	24	Whole proteome	169	[31]
Enteric autoimmune diseases	32	Immunoepitopes	58	[36]
Nervous system	40	Spike	8	[34]
Neuropathies	41	Whole proteome	2	[30]
Pulmonary surfactant	92	Spike	13	[21]

^∗^All epitopes from the SARS-CoV-2 proteome are validated as immunogenic in the IEDB.

**Table 2:** Bioinformatic studies addressing mimicry between SARS-CoV-2 and human proteins analyzing the whole human proteome.

Human proteins	SARS-CoV-2 proteins	Shared peptides	Reference
Whole proteome	Whole proteome	3781	[25]
	Whole proteome	33	[26]
	Spike	356	[27]
	Immunoepitopes	54	[28]
	Immunoepitopes	230	[19]

Immunoepitopes^∗^	Spike	18	[32]

^∗^All epitopes from human or SARS-CoV-2 proteome are validated as immunogenic in the IEDB.

## Data Availability

Data sharing is not applicable to this article as no datasets were generated or analyzed during the current study.
